# Reinforcement Learning for Routing in Cognitive Radio Ad Hoc Networks

**DOI:** 10.1155/2014/960584

**Published:** 2014-07-16

**Authors:** Hasan A. A. Al-Rawi, Kok-Lim Alvin Yau, Hafizal Mohamad, Nordin Ramli, Wahidah Hashim

**Affiliations:** ^1^Department of Computer Science and Networked Systems, Sunway University, No. 5 Jalan Universiti, Bandar Sunway, 46150 Petaling Jaya, Selangor, Malaysia; ^2^Wireless Network and Protocol Research Lab, MIMOS Berhad, Technology Park Malaysia, 57000 Kuala Lumpur, Malaysia

## Abstract

Cognitive radio (CR) enables unlicensed users (or secondary users, SUs) to sense for and exploit underutilized licensed spectrum owned by the licensed users (or primary users, PUs). Reinforcement learning (RL) is an artificial intelligence approach that enables a node to observe, learn, and make appropriate decisions on action selection in order to maximize network performance. Routing enables a source node to search for a least-cost route to its destination node. While there have been increasing efforts to enhance the traditional RL approach for routing in wireless networks, this research area remains largely unexplored in the domain of routing in CR networks. This paper applies RL in routing and investigates the effects of various features of RL (i.e., reward function, exploitation, and exploration, as well as learning rate) through simulation. New approaches and recommendations are proposed to enhance the features in order to improve the network performance brought about by RL to routing. Simulation results show that the RL parameters of the reward function, exploitation, and exploration, as well as learning rate, must be well regulated, and the new approaches proposed in this paper improves SUs' network performance without significantly jeopardizing PUs' network performance, specifically SUs' interference to PUs.

## 1. Introduction

Cognitive radio (CR) has been proposed to enable unlicensed users (or secondary users, SUs) to exploit the underutilized licensed channels (or white spaces) owned by the licensed users (or primary users, PUs). Most traditional routing schemes adopt a rule-based approach [1] in which each node keeps and follows a set of predefined rules in its action selection for different network conditions; and this may not suit CR due to its intrinsic characteristic of the* dynamicity* and* unpredictability *of the network conditions (i.e., PUs' activities and channel quality) which require context awareness and intelligence [2–4]. Context awareness enables a SU node to observe the operating environment; while intelligence enables the SU node to learn and make action selection that maximizes network performance as time goes by. These capabilities are essential as the rule-based approach may not be feasible to define actions for all possible sets of network conditions in CR networks (CRNs).

Reinforcement learning (RL) [5], which is an artificial intelligence approach, has been applied to achieve context awareness and intelligence in CRNs [2]. This article presents a simulation study on the application of RL to routing in CRNs. Firstly, the traditional RL approach is applied in a routing scheme, which we call Cognitive Radio Q-routing (CRQ-routing). Next, a RL feature, namely reward function, is investigated. An enhanced RL-based routing scheme called weighted cognitive radio Q-routing (WCRQ-routing) is proposed. Subsequently, other two RL features, namely, exploitation and exploration, as well as learning rate, are investigated. The network performance of RL-based routing schemes can be enhanced by regulating the RL features; hence, new enhancements are proposed for the reward function, exploitation, and exploration, as well as learning rate.

CRQ-routing is a spectrum-aware scheme that finds least-cost routes in CRNs taking into account the dynamicity and unpredictability of the channel availability and channel quality. Simulation results show that CRQ-routing and its enhancements minimize SUs' interference to PUs, SUs' end-to-end delay, and SUs' packet loss rate, as well as maximizing SUs' throughput.

Our contributions are as follows.

- Section 3 presents CRQ-routing which applies the traditional RL approach.
- Section 4 investigates different reward representations for network performance enhancement. In addition to CRQ-routing, this section investigates a variant of the reward function which we conveniently call WCRQ-routing. WCRQ-routing applies a weight factor *ω* to adjust the tradeoff between PUs' and SUs' network performance. Performance enhancement achieved by CRQ-routing and WCRQ-routing for different PU utilization levels (PULs) and packet error rate (PER) are compared with the traditional shortest path (SP) routing scheme and the optimal primary user-aware shortest path (PASP) routing scheme.
- Section 5 investigates the effects of exploitation and exploration on network performance. A simple and pragmatic exploration approach called dynamic softmax (DS) is proposed to dynamically regulate the frequency of exploration according to the dynamicity of the operating environment. Performance enhancement achieved by DS is compared with two traditional exploration approaches, namely, *ε*-greedy and softmax.
- Section 6 investigates the effects of learning rate on network performance. A simple and pragmatic learning rate adjustment approach called the counterapproach (CA), which is based on the traditional win-or-learn-fast policy hill climbing (or win-lose) [6], is proposed to dynamically regulate the learning rate according to the dynamicity of the operating environment. Performance enhancement achieved by CA is compared with the traditional approach, namely, win-lose.

Simulation experiment, results, and discussions are presented in each of the sections. Finally, Section 7 concludes this paper.

## 2. Related Work

While most researches focus on the enhancement of either PUs' or SUs' network performance [7–10], this paper focuses on both PUs' and SUs'. CRQ-routing minimizes SUs' interference to PUs without causing significant detrimental effects on SUs' network-wide performance.

In [7–10], either one or both of the following requirements are applicable. Firstly, information on PUs' and SUs' physical locations is essential. The associated challenges are additional energy consumption, increased hardware cost, and the availability of the physical location information in indoor scenarios [2]. Secondly, network-wide information such as link cost is essential; however, it is difficult to obtain up-to-date information for the entire network in the presence of dynamicity and unpredictability of the channel availability and channel quality in CRNs. For instance, a reactive routing scheme requires a SU destination node to confirm a route prior to data transmission; however, due to the dynamicity and unpredictability of the channel availability and channel quality, a new route may have expired before routing information reaches the SU destination node. CRQ-routing does not require geographical and network-wide information, and it adopts a per-hop routing approach (rather than an end-to-end routing approach) that enables each SU intermediate node to make routing decision for a single hop to its next-hop node based on local information.

The application of RL to routing schemes in CRNs has been limited, such as [11], although it has been shown to improve routing performance in various traditional wireless networks [12, 13]. In [11], the SUs' network performance is shown to be enhanced, while in CRQ-routing, both PUs' and SUs' network performances are enhanced. Using RL, CRQ-routing integrates route discovery mechanism with channel selection. CRQ-routing is a multipath routing scheme that enables a SU node to maintain multiple routes, and this can be well incorporated into RL through its feature called exploration. Generally speaking, the existence of multiple routes helps to enhance network reliability and to achieve load balancing among various routes. This is because a SU can automatically switch its route to another one during route recovery in the event of route failure.

## 3. CRQ-Routing: Application of the Traditional Reinforcement Learning Approach to Routing

This section presents CRQ-routing that takes account of the PUs' and SUs' network performance by minimizing SUs' interference to PUs along a route without significantly jeopardizing SUs' network-wide performance. It applies a traditional RL approach called Q-learning [14, 15], which is a popular RL approach. CRQ-routing enables a SU to observe its local operating environment regularly and subsequently to learn an action selection policy through exploring various routes, and finally to choose routes with enhanced network performance (i.e., lower SUs' interference to PUs, lower SUs' end-to-end delay, lower SUs' packet loss rate, and higher SUs' throughput). Generally speaking, RL enables a SU node to

- estimate the dynamic link cost. This allows a SU to learn about and adapt to the local network conditions (i.e., PUs' activities and channel quality) which are dynamic and unpredictable in nature,
- search for the best-possible route using information observed from the local operating environment and information received from neighboring nodes,
- incorporate a wide range of factors that can affect the routing performance into consideration, including both PUs' activities and channel quality.

By choosing links with lower link-layer delay, which is the time duration required to deliver a SU's packet to a next-hop node successfully, SUs' interference to PUs can be reduced and SUs' end-to-end network performance can be enhanced. Note that the link-layer delay includes the time duration incurred by retransmission as a result of PU-SU packet collisions. The RL model for CRQ-routing is shown in Table 1, and it is embedded in a SU node *i*. Using CRQ-routing, each SU chooses a next-hop node and channel pair as part of a route. There are three key representations for the RL model as follows.

- State *s*_*t*_^*i*^ ∈ *S* = {1, 2, …, *N* − 1} represents a SU destination node *n*, where *N* represents the number of SUs in the entire network.
- Action *a*_*t*_^*i*^ ∈ *A* ^*i*^ = {1, 2, …, *J*} represents the selection of a next-hop SU neighbor node *j* along with its operating channel, where *J* represents the number of SU *i*'s neighboring SU nodes.
- Cost *r*_*t*_^*i*^(*a*_*t*_^*i*^), which indicates the consequence upon taking action *a*_*t*_^*i*^, represents the link-layer delay of a SU communication node pair, namely nodes *i* and *j*. The link-layer delay includes retransmission delays caused by packet loss and PU-SU packet collision. Hence, the end-to-end delay (or the accumulated link-layer delay) reflects the accumulated SUs' interference to PUs; and by achieving lower delay, the interference level can be reduced.

Each SU node *i* keeps track of *Q*-value *Q*_*t*_^*i*^(*s*_*t*_^*i*^, *a*_*t*_^*i*^), which relates the three representations, in its *Q*-table (i.e., routing table). For each state-action pair, the *Q*-value represents the accumulated link-layer delay of a route leading to SU destination node *s*_*t*_^*i*^ by choosing a SU next-hop node *a*_*t*_^*i*^ = *j*. At time *t*, SU *i* selects a SU next-hop node *a*_*t*_^*i*^ = *j* as part of a route to reach destination node *s*_*t*_^*i*^; and upon a successful transmission at time *t* + 1, it receives an end-to-end delay estimate for the route, namely *Q*_*t*_^*j*^(*s*_*t*_^*j*^, *k*), from node *j* and estimates the link-layer delay *r*_*t*+1_^*i*^(*j*). Note that the link-layer delay is dynamic and unpredictable due to the nature of the PUs' activities in which there are different levels of PUL. In general, the link-layer delay increases with PUL in a channel. The *Q*-value *Q*_*t*_^*i*^(*s*_*t*_^*i*^, *a*_*t*_^*i*^ = *j*) is updated as follows:

(1) Qt+1i(sti,j)⟵(1−α)Qti(sti,j) +α(rt+1i(j)+min⁡k∈Aj⁡Qtj(stj,k)),

where 0 ≤ *α* ≤ 1 is the learning rate and node *k* ∈ *A* ^*j*^ is an upstream node of SU node *j*. A SU node *i* adopts a policy *π*_*t*+1_^*i*^(*s*_*t*_^*i*^) that chooses a SU next-hop node with the minimum cost as follows:

(2) $$\begin{array}{c} \pi_{t+1}^{i}\left(s_{t}^{i}\right)=\underset{a\;\in \;A^{i}}{\text{argmin}}\left(Q_{t}^{i}\left(s_{t}^{i},a\right)\right). \end{array}$$

In the next section, we present a variant of the reward representation in RL to further enhance PUs' and SUs' network performance.

## 4. WCRQ-Routing: A Variant of the Reward Representation

WCRQ-routing incorporates a weight factor into the reward representation of CRQ-routing to adjust the tradeoff between PUs' and SUs' network performance. WCRQ-routing provides further enhancement on SUs' network performance without jeopardizing PUs' network performance. The main difference between WCRQ-routing and CRQ-routing is the cost (or negative reward) representation as follows.

- CRQ-routing enables a SU to learn about the accumulated cost in terms of link-layer delay along a route.
- WCRQ-routing enables a SU to learn about the accumulated cost in terms of the number of SUs' packet retransmissions and the packet queue length of SUs along a route. Hence, WCRQ-routing enables a SU to take account of packet retransmission which further improves the PUs' network performance, and network performance which further improves the SUs' network performance. WCRQ-routing also incorporates a weight factor *ω* which adjusts the tradeoff between PUs' and SUs' network performance.

The RL model for WCRQ-routing is shown in Table 2, and it is embedded in a SU node *i*. The state and action representations are similar for CRQ-routing (see Table 1) and WCRQ-routing, and so only the reward representation is shown. The reward representation for the RL model is as follows.

- Cost *r*_*t*_^*i*^(*a*_*t*_^*i*^) = *ωr*_*t*_^*i*,*j*^ + (1 − *ω*)*q*_*t*_^*j*^ has two components: (1)  *r*_*t*_^*i*,*j*^ represents the number of retransmissions for a packet sent from SU node *i* to SU neighbor node *j* at time *t* as a result of PU-SU packet collisions and SU packet loss and (2)  *q*_*t*_^*j*^ represents the number of packets in the queue of SU neighbor node *j*. Both *r*_*t*_^*i*,*j*^ and *q*_*t*_^*j*^ values in reward *r*_*t*_^*i*^(*a*_*t*_^*i*^) are normalized to [0,1]. The weight factor *ω* = [0,1] adjusts the tradeoff between PUs' and SUs' network performance.

Similar to CRQ-routing, each SU node *i* keeps track of *Q*-values *Q*_*t*_^*i*^(*s*_*t*_^*i*^, *a*_*t*_^*i*^) in its *Q*-table (i.e., routing table). For each state-action pair, the *Q*-value represents a weighted cost that takes account of the number of packet retransmissions and packet queue length of SUs along a route leading to SU destination node *s*_*t*_^*i*^ by choosing a SU next-hop node *a*_*t*_^*i*^. At time *t*, SU *i* selects a SU next-hop node *a*_*t*_^*i*^ = *j* as part of a route to reach destination node *s*_*t*_^*i*^ and upon a successful transmission, it receives an estimate of the weighted cost for the route, namely *Q*_*t*_^*j*^(*s*_*t*_^*j*^, *k*), from node *j* and estimates reward *r*_*t*+1_^*i*^(*j*), which takes into account the number of retransmissions for a packet and the number of packets in the queue of SU neighbor node *j* at time *t* + 1. Note that both number of retransmissions for a packet and number of packets in the queue of a SU's neighbor node are dynamic and unpredictable due to the nature of the PUs' activities in which there are different levels of PUL. In general, *r*_*t*+1_^*i*^(*j*) increases with PUL in a channel. The *Q*-value *Q*_*t*_^*i*^(*s*_*t*_^*i*^, *a*_*t*_^*i*^) is updated using (1) and a SU next-hop node with the minimum cost is chosen using (2).

The rest of this section is organized as follows.  Section 4.1 presents simulation setup and parameters.  Section 4.2 presents a comparison of network performance achieved by CRQ-routing, WCRQ-routing, and two baseline routing schemes, namely, shortest path (SP) and PU aware shortest path (PASP) routing schemes.  Section 4.3 presents the effects of the weight factor *ω* in reward representation on network performance.

### 4.1. Simulation Setup and Parameters

**Figure 1:**
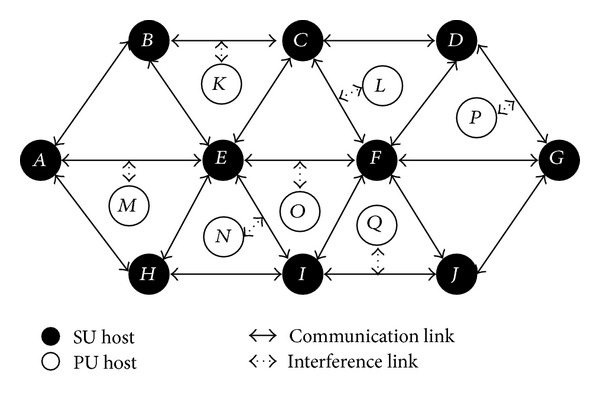
Network scenario.

Figure 1 shows the system model which is a multihop CRN with *N* SUs and *K* PUs [16]. Each PU transmits in one of the *K* different channels. In Figure 1, we consider a SU source node *A* and a SU destination node *G*.

We compare the network performance achieved by CRQ-routing and WCRQ-routing with nonlearning approaches, specifically, the traditional SP routing and the optimal PASP routing approaches. SP routing selects a route that has the minimum number of hops and this has been shown to improve the end-to-end network performance in traditional networks. PASP routing selects a route that has the minimum accumulated amount of PUs' activities. This means that the route encounters the least number of PUs, and so the PUL along the route may be the lowest. However, PASP routing may not be feasible in practice as it is a centralized approach that requires network-wide information of PUL for each link and channel. Nevertheless, PASP routing is an optimal approach that minimizes SUs' interference to PUs and so it serves as a good comparison in our simulation study.

There are four network performance metrics as follows.

- *PU-SU collision probability* indicates the level of SUs' interference to PUs. Reducing this metric improves the PUs' network performance. This metric is a ratio of the number of PU-SU collisions to the number of SUs' packet transmissions.
- *SU end-to-end delay* includes the transmission, processing, backoff, and queuing delays along a route.
- *SU packet loss rate* is a ratio of the number of packet loss to the total number of packets sent.
- *SU throughput* is the number of arriving packets per second (pps) at the SU destination node.

The simulation compares the aforementioned performance metrics with respect to different levels of PULs for CRQ-routing, WCRQ-routing, SP routing, and PASP routing. The dynamicity of the PUs' activities (or PUL) is represented by PU arrival rate *μ*_PUL_, and each scenario has a certain level of unpredictability of the PUs' activities (or the standard deviation of PU arrival rate) which is represented by *σ*_PUL_.

Table 3 shows a summary of the simulation parameters and values. Generally speaking, each simulation is run for 100 seconds and repeated 50 times with different random seeds. The simulation reporting interval is 1 second which indicates that, at each second of the simulation running time, a mean value of the simulation result is calculated. The default number of SUs is *N* = 10, and PUs is *K* = 19. Each PU does not change its channel and operates in distinctive channels. Each PU activity in channel *k* ∈ *K* is modeled as a Poisson process with mean *λ*_PUL_^*k*^(*μ*_PUL_, *σ*_PUL_), which is assigned using Box-Muller transform [17] according to an expected mean *μ*_PUL_ = [0, 1] and standard deviation *σ*_PUL_ = [0, 1]. The standard deviation of PUL is *σ*_PUL_ ∈ {0.0, 0.4, 0.8}, which indicates low, medium, and high levels of unpredictability of the PUs' activities, and these values are chosen due to their significant effects to the results. Since the focus is on the comparison of network performance in regard to PUL, we assume the channels have low level of noise with *μ*_PER_ = 0.05 and *σ*_PER_ = 0.025. Packets are generated at the SU source node using Poisson process with a mean arrival rate of *λ*_SU_ = 0.6. Since the PUs have higher priority than SUs, their transmissions take longer; specifically, the SUs' transmission delay is *d*_SU_^tr⁡^ = 1.0 ms and the PUs' transmission delay is *d*_PU_^tr⁡^ = 1.2 ms. In order to model a simple queuing delay *d*_SU_^qu^, we assume a finite packet queue size of 1000 packets in each SU node with a constant processing delay *d*_SU_^pr^ = 1.0 ms. The *Q*-values are initialized to 0 in order to encourage exploration at the start of the simulation. The SU learning rate is *α* = 0.5 (see (1)). For WCRQ-routing, the weight factor is set to *ω* = 0.5 so that there is a balanced tradeoff between PUs' and SUs' network performance. The effects of *ω* on the network performance are presented in Section 4.3.

### 4.2. Comparison of CRQ-Routing, WCRQ-Routing, SP, and PASP Routing Schemes

We present simulation results for the four performance metrics in this section.

#### 4.2.1. SUs' Interference to PUs

**Figure 2:**
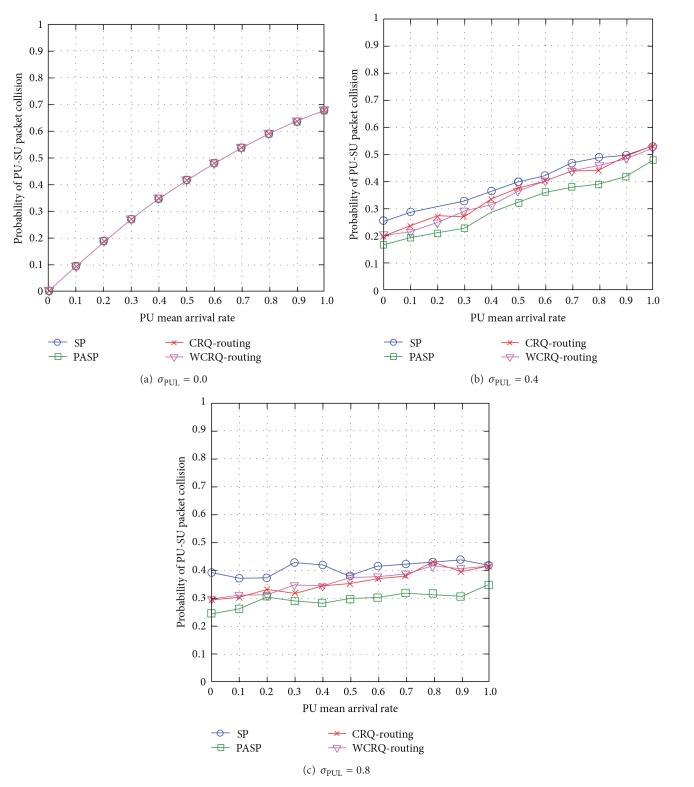
SUs' interference to PUs for varying PU mean arrival rate *μ*_PUL_ for different levels of standard deviation of PUL *σ*_PUL_.

When the standard deviation of PUL is low *σ*_PUL_ = 0, most next-hop node (or link) and channel pairs have the same PU mean arrival rate *μ*_PUL_, so all routing schemes achieve similar probability of PU-SU packet collisions across a CRN (see Figure 2(a)). When the unpredictability level of PUL is *σ*_PUL_ = 0.4, the link and channel pairs have greater difference in the levels of PU mean arrival rate *μ*_PUL_, WCRQ-routing, and CRQ-routing choose routes that minimize SUs' interference to PUs. Both WCRQ-routing and CRQ-routing reduce collisions with PUs for up to 19% compared to SP routing, whereas the empirical PASP routing scheme, which provides the best results, reduces collisions with PUs for up to 30% compared to SP routing (see Figure 2(b)). When *σ*_PUL_ = 0.8, the link and channel pairs have the greatest difference in the levels of PU mean arrival rate *μ*_PUL_. Similar trends to the network scenario of *σ*_PUL_ = 0.4 are observed although collisions with PUs have generally increased due to the increased unpredictability of PUs' activities (see Figure 2(c)).

Generally speaking, both WCRQ-routing and CRQ-routing achieves almost similar performance in terms of SUs' interference to PUs; and the SUs' interference to PUs increases with the PU mean arrival rate *μ*_PUL_ and the standard deviation of PUL *σ*_PUL_.

#### 4.2.2. SU End-to-End Delay

**Figure 3:**
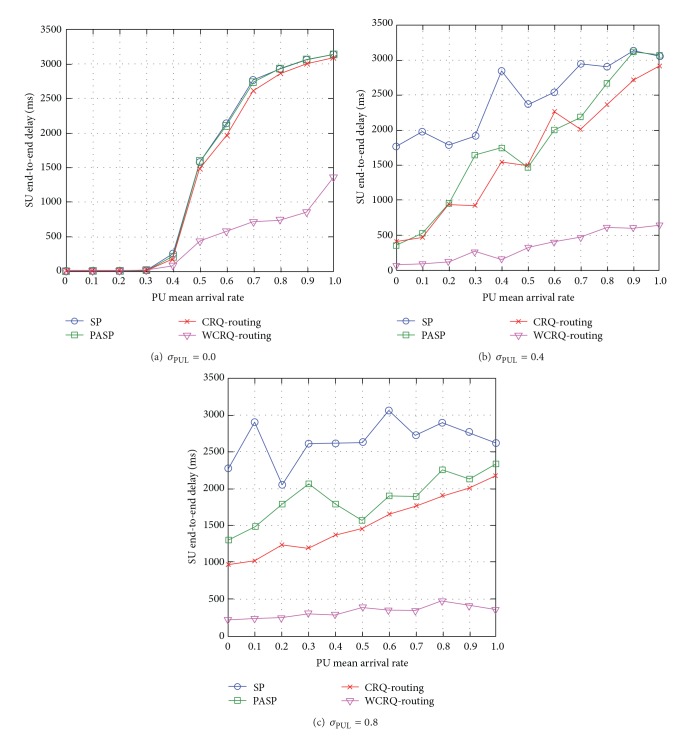
SU end-to-end delay for varying PU mean arrival rate *μ*_PUL_ for different levels of standard deviation of PUL *σ*_PUL_.

When the standard deviation of PUL is low *σ*_PUL_ = 0, most link and channel pairs have the same PU mean arrival rate *μ*_PUL_. The SU end-to-end delay increases with PU mean arrival rate *μ*_PUL_ (see Figure 3(a)). When the PU mean arrival rate *μ*_PUL_ is low, the SU end-to-end delay consists of mainly transmission and queuing delays, and when *μ*_PUL_ becomes higher (i.e., *μ*_PUL_ > 0.3), the retransmission and backoff delays caused by PU-SU packet collisions and overflow of SUs' packet queues increase contributing to higher SU end-to-end delay. WCRQ-routing reduces SU end-to-end delay for up to 72% compared to the other routing schemes. This is because WCRQ-routing takes into account the queue length of SUs along the route, so it chooses routes with lower network congestion contributing to lower SU end-to-end delay.

When the unpredictability level of PUL increases from *σ*_PUL_ = 0.4 to *σ*_PUL_ = 0.8, the link and channel pairs have greater difference in the levels of PU mean arrival rate *μ*_PUL_. WCRQ-routing chooses routes that minimize the number of packet retransmissions caused by PU-SU packet collisions and overflow of SUs' packet queues contributing to lower SU end-to-end delay (see Figures 3(b) and 3(c)). With greater unpredictability level of PUL *σ*_PUL_, it is easier for WCRQ-routing to find a route with better performance; hence, the lower SU end-to-end delay when *σ*_PUL_ = 0.8 in Figure 3(c) compared to *σ*_PUL_ = 0.4 in Figure 3(b), and WCRQ-routing achieves lower SU end-to-end delay for up to 89% when *σ*_PUL_ = 0.8 compared to other routing schemes. Additionally, there are two main observations. Firstly, the fluctuations of SU end-to-end delay are observed because the routes of SP routing and PASP routing are static as they are unaware of the unpredictability of PUL, while the routes of CRQ-routing and WCRQ-routing are dynamic in nature. Secondly, PASP routing and CRQ-routing deteriorate to the network performance of SP routing with increasing PU mean arrival rate *μ*_PUL_ because both schemes take account of PU mean arrival rate *μ*_PUL_ only in routing decision, and so when *μ*_PUL_ becomes higher, they may choose longer routes resulting in higher SU end-to-end delay. In contrast, WCRQ-routing takes account of network congestion as well.

Generally speaking, WCRQ-routing reduces SU end-to-end delay, and the metric increases with the PU mean arrival rate *μ*_PUL_ and reduces with the standard deviation of PUL *σ*_PUL_.

#### 4.2.3. SU Packet Loss

**Figure 4:**
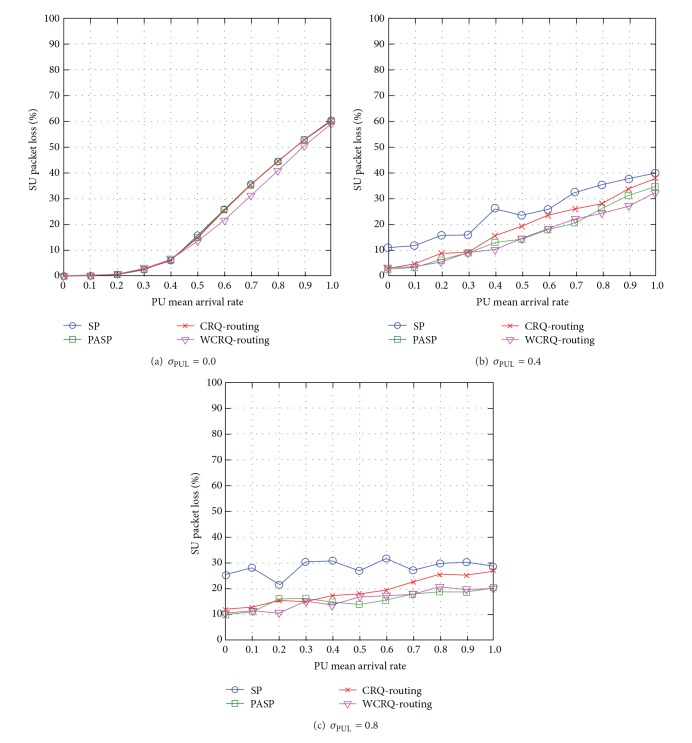
SU packet loss for varying PU mean arrival rate *μ*_PUL_ for different levels of standard deviation of PUL *σ*_PUL_.

When the standard deviation of PUL is low *σ*_PUL_ = 0, WCRQ-routing achieves load-balancing among the available routes as it takes into account the queue length of SUs along a route, so it reduces network congestion, and hence there is lower SU packet loss of up to 16% compared to the other routing schemes (see Figure 4(a)).

When the unpredictability level of PUL is *σ*_PUL_ = 0.4 and *σ*_PUL_ = 0.8, WCRQ-routing achieves almost similar network performance to PASP routing with lower packet loss for up to 56% compared to SP routing and 25% compared to CRQ-routing when *σ*_PUL_ = 0.8 (see Figures 4(b) and 4(c)). This is because WCRQ-routing chooses routes with lower network congestion in order to minimize packet retransmission caused by PU-SU packet collisions and overflow of SU's packet queues leading to lower number of packet retransmissions. Both network scenarios of *σ*_PUL_ = 0.4 and *σ*_PUL_ = 0.8 share almost similar trends although the SU packet loss has generally decreased in the case of *σ*_PUL_ = 0.8.

Generally speaking, WCRQ-routing reduces SU packet loss, and the metric increases with the PU mean arrival rate *μ*_PUL_ and reduces with the standard deviation of PUL *σ*_PUL_.

#### 4.2.4. SU Throughput

**Figure 5:**
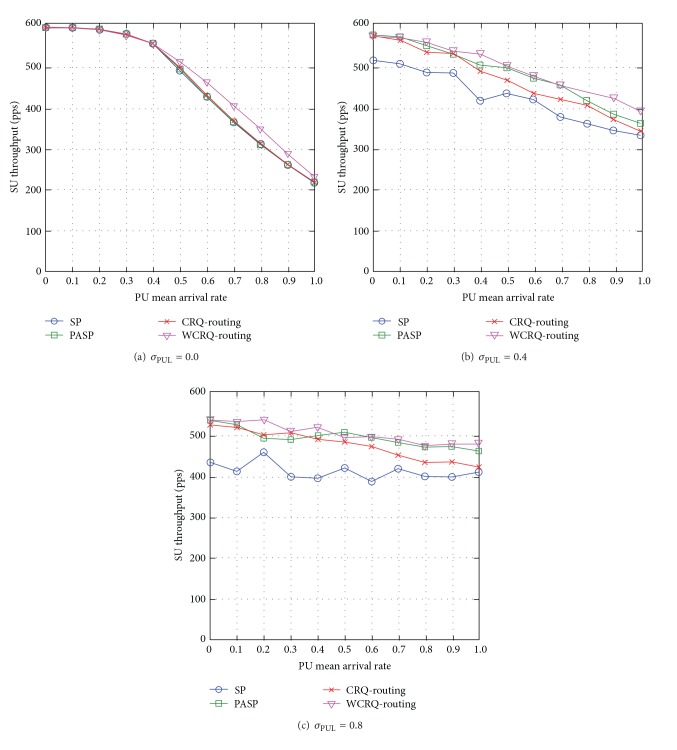
SU throughput for varying PU mean arrival rate *μ*_PUL_ for different levels of standard deviation of PUL *σ*_PUL_.

When the standard deviation of PUL is low *σ*_PUL_ = 0, WCRQ-routing achieves load-balancing among available routes as it takes into account the queue length of SUs along a route, so it reduces network congestion, and hence there is higher SU throughput of up to 11% compared to the other routing schemes (see Figure 5(a)).

When the unpredictability level of PUL is *σ*_PUL_ = 0.4 and *σ*_PUL_ = 0.8, WCRQ-routing achieves higher SU throughput of up to 27% compared to SP routing, up to 12% compared to CRQ-routing, and up to 9% compared to PASP routing when *σ*_PUL_ = 0.8 (see Figures 5(b) and 5(c)). This is because WCRQ-routing chooses routes with lower network congestion in order to minimize packet retransmission caused by PU-SU packet collisions and overflow of SU's packet queues leading to higher SU throughput.

Generally speaking, WCRQ-routing increases SU throughput, and the metric reduces with the PU mean arrival rate *μ*_PUL_ and reduces with the standard deviation of PUL *σ*_PUL_.

#### 4.2.5. Section Summary

We summarize simulation outcomes for the comparison of CRQ-routing, WCRQ-routing, SP, and PASP routing schemes as follows.

- Network performance degrades as the dynamicity of PUs' activities *μ*_PUL_ increases.
- WCRQ-routing and CRQ-routing minimize SUs' interference to PUs with respect to PUL in the presence of dynamicity and unpredictability of the channel availability.
- WCRQ-routing enhances network performance of SUs compared to other routing schemes including lower SU end-to-end delay, lower SU packet loss, and higher SU throughput. Using a weight factor in WCRQ-routing, the cost represents two factors including the number of packet retransmissions and the packet queue length of SUs.

### 4.3. Effects of Weight Factor in Reward Representation

This section investigates the effects of the weight factor *ω* of WCRQ-routing on network performance. WCRQ-routing applies a weight factor *ω* to adjust the tradeoff between PUs' and SUs' network performance. Based on Table 2, with a higher value of *ω*, there is greater consideration on *r*_*t*_^*i*,*j*^ which represents the number of retransmissions for a packet sent from SU node *i* to SU neighbor node *j* at time *t* and indicates the probability of PU-SU packet collision, and lesser consideration on SU neighbor node *j*'s queue length *q*_*t*_^*j*^ which indicates SU network congestion. This means a higher value of weight factor *ω* improves the PUs' network performance, while a lower value of *ω* improves the SUs' network performance.

While Table 3 presents the default simulation parameters and values, Table 4 presents the specific simulation parameters and values for this investigation. Generally speaking, we simplify the simulation values in order to focus on the effects of weight factor *ω*. This explains why we consider two different PUL *μ*_PUL_ and PER *μ*_PER_ values only and a fixed value of the standard deviation of PUL *σ*_PUL_. We increase the network congestion level with increased SU mean arrival rate to *λ*_SU_ = 0.8.

In this section, we assume that when PU-SU packet collision occurs, a SU packet is transmitted successfully while the PU's packet is lost; therefore, the PUs' activities can be easily affected and it is prone to SUs' interference. We present the simulation results for the four performance metrics in the rest of this section.

#### 4.3.1. SUs' Interference to PUs

**Figure 6:**
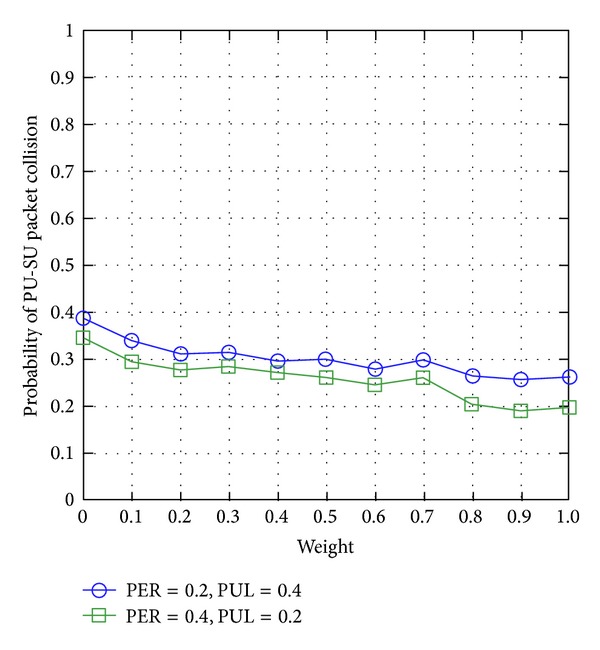
SUs' interference to PUs for varying weight factor *ω*.

Figure 6 shows that the SUs' interference to PUs decreases with the weight factor *ω* of WCRQ-routing. Hence, a higher value of weight factor *ω* improves the PUs' network performance, while a lower value of *ω* improves SUs' network performance. In addition, with respect to PUL and PER, Figure 6 shows that PUL has greater effects on PUs' network performance compared to PER because PUL has a direct effect on the PU-SU packet collisions; hence, higher PULs indicate higher probability of PU-SU collisions.

#### 4.3.2. SU End-to-End Delay

**Figure 7:**
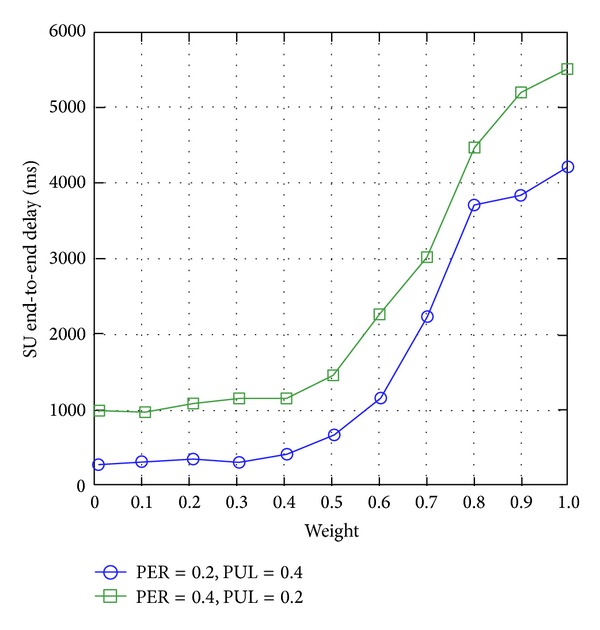
SU end-to-end delay for varying weight factor *ω*.

Figure 7 shows that the SU end-to-end delay increases with the weight factor *ω* of WCRQ-routing. Hence, a higher value of weight factor *ω* improves the PUs' network performance; while a lower value of *ω* improves SUs' network performance. In addition, with respect to PUL and PER, Figure 7 shows that PER has greater effects on SUs' network performance compared to PUL because PER has a direct effect on the packet length and a SU's packet can be transmitted successfully during a PU-SU packet collision. With a higher value of PER (or a noisier channel), SU network congestion increases due to increasing number of SU packet retransmissions leading to higher SU end-to-end delay.

#### 4.3.3. SU Packet Loss

**Figure 8:**
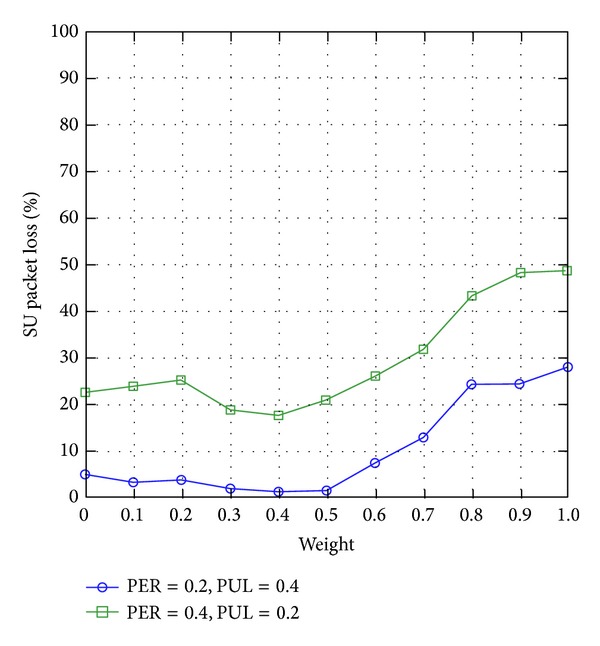
SU packet loss for varying weight factor *ω*.

Figure 8 shows that the SU packet loss increases with the weight factor *ω* of WCRQ-routing. The explanations leading to this circumstance are similar to those found in the investigation of SU end-to-end delay (see Section 4.3.2). Interestingly, Figure 8 shows that when weight factor is *ω*≅0.5, WCRQ-routing achieves the lowest packet loss. At *ω* = 0.5, a SU node *i* gives equal consideration to both link quality *r*_*t*_^*i*,*j*^, which aims to avoid routes with higher PUs' activities in order to reduce SUs' backoff delays, and SU *j*'s queue length *q*_*t*_^*j*^, both of which reduce the overflow of SUs' queues and SUs' packet loss.

#### 4.3.4. SU Throughput

**Figure 9:**
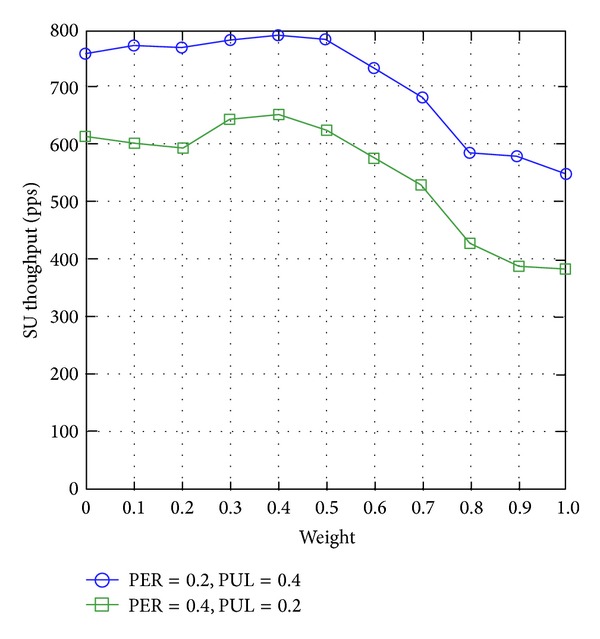
SU throughput for varying weight factor *ω*.

Figure 9 shows that the SU throughput reduces with the weight factor*ω* of WCRQ-routing. The explanations leading to this circumstance are similar to those found in the investigation of SU end-to-end delay (see Section 4.3.2); and when weight factor is *ω*≅0.5, WCRQ-routing achieves the highest throughput.

#### 4.3.5. Section Summary

We summarize simulation outcomes for the effects of weight factor in reward representation as follows.

- A higher value of weight factor *ω* improves the PUs' network performance; while a lower value of *ω* improves the SUs' network performance.
- A balanced weight factor *ω*≅0.5 achieves the best-possible SUs' network performance, particularly lower SU packet loss and higher SU throughput.

## 5. Enhancement of Exploration Mechanism

Traditionally, during route selection, there are two types of actions, namely, exploitation and exploration. Exploitation selects the best-known route *a*_*t*_^*i*^ = argmin_*a*∈*A*_ *Q*_*t*_^*i*^(*s*_*t*_^*i*^, *a*), which has the lowest cost, in order to improve network performance. Exploration selects a random route *a*_*t*_^*i*^ ∈ *A* in order to improve knowledge, specifically, the estimation of *Q*-values for various routes. Two traditional exploration schemes are *ε*-greedy and softmax [5], and these schemes have been applied to regulate the exploration probability. A well-balanced tradeoff between exploitation and exploration helps to maximize network performance as time goes by. There have been some limited efforts to investigate this tradeoff in wireless networks; and this investigation applies to routing in CRNs. We present an overview of exploration probability and the traditional (called *ε*-greedy and softmax) and our proposed (called dynamic softmax) exploration approaches to dynamically regulate the exploration probability, as well as simulation results for the four performance metrics, in the rest of this section.

### 5.1. An Overview of Exploitation and Exploration

While route exploitation may seem to improve network performance as it forwards SUs' packets using the best-known route, it may cause network congestion and subsequently degrade SUs' network performance. On the other hand, route exploration reduces traffic load on the best-known route, and so it may increase the convergence rate to an optimal route in a dynamic and unpredictable operating environment. However, if the exploration probability is high, forwarding the SUs' packets along nonoptimal routes may degrade SUs' network performance causing higher end-to-end delay and packet loss, as well as lower throughput.

**Figure 10:**
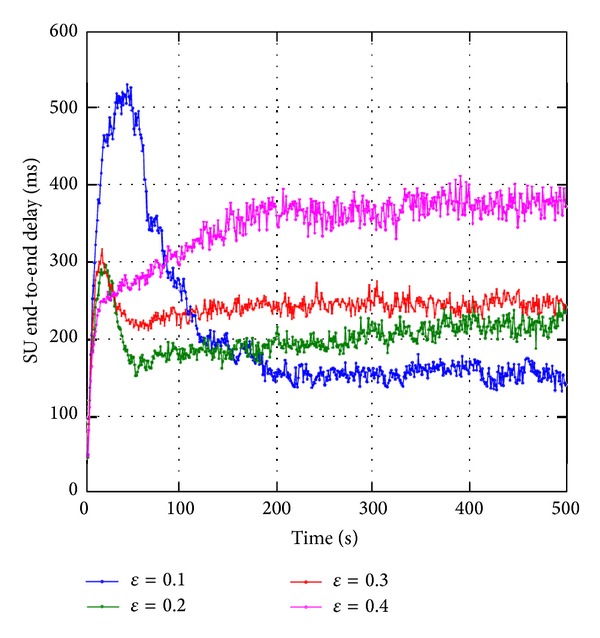
SU end-to-end delay for varying exploration probability *ε*.

We present simulation results of a preliminary investigation to show the effects of exploration probability *ε* on the convergence rate of SUs' network performance, particularly SU end-to-end delay. Note that more details on simulation setup and parameters are presented in later section. Using *ε*-greedy, a SU explores with a small probability *ε* and exploits with probability 1 − *ε*. Convergence rate is the time duration for a SU node to find an optimal or near-optimal route, which provides a stable and enhanced SUs' network performance.  Figure 10 shows that, as the exploration probability increases, the convergence rate increases (e.g., from approximately 200 seconds using *ε* = 0.1 to 100 seconds using *ε* = 0.2). The average SU end-to-end delay increases with the exploration probability *ε*. Additionally, higher exploration probability causes instability of SUs' network performance (e.g., SU end-to-end delay has higher fluctuations using *ε* = 0.4). Also, a peak is observed when exploration probability is low using *ε* = 0.1 because a SU may exploit nonoptimal routes at most of the time since the* Q*-values are initialized to 0 values which encourages exploration.

This initial investigation motivates us to achieve a balanced tradeoff between exploitation and exploration in route selection.

### 5.2. Exploration Approaches

We present two traditional exploration approaches (i.e., *ε*-greedy and softmax) and a variant of the exploration approach (i.e., dynamic softmax) in this section.

#### 5.2.1. The *ε*-Greedy Approach

The *ε-*greedy approach performs exploration with a small probability *ε* (e.g., *ε* = 0.1) and exploitation with probability 1 − *ε* [5]. A drawback is that, during exploration, it selects nonoptimal routes with below-average performance in a random manner with equal probability, and if these routes are chosen most of the times, the SUs' network performance may degrade.

#### 5.2.2. The Softmax Approach

The softmax approach chooses an exploration action based on* Q*-values [5]. Specifically, routes with lower* Q*-values (i.e., lower cost) are likely to be explored than routes with higher* Q*-values (i.e., higher cost), and this addresses the drawback of *ε*-greedy; specifically, it minimizes the detrimental effects of exploration to SUs' network performance while exploring nonoptimal routes.

Using softmax (or the Boltzmann distribution), SU node *i* chooses its next-hop SU neighbor node *a*_*t*_^*i*^ ∈ *A* with the following probability:

(3) $$\begin{array}{c} P\left(s_{t}^{i},a_{t}^{i}\right)=\;\frac{e^{-Q_{t}^{i}(s_{t}^{i},a_{t}^{i})/M}}{\sum_{a\;\in \;A^{i}}e^{-Q_{t}^{i}(s_{t}^{i},a)/M}}, \end{array}$$

where *A* ^*i*^ represents a set of SU node *i*'s neighbor nodes and *M* is the temperature that determines the level of exploration. Higher *M* value indicates higher possibility of exploration, whereas lower *M* value indicates higher possibility of exploitation.

#### 5.2.3. The Dynamic Softmax Approach

In *ε*-greedy and softmax, the exploration probability is predefined; specifically, the exploration probability *ε* and tempreture *M* are predefined. There are two shortcomings. Firstly, while the optimal *ε* and *M* values are dependent on the dynamicity and unpredictability of the network conditions, it may not be feasible to determine these values on the fly. Secondly, routing in CRNs involves a number of SU nodes, and it may not be feasible to determine the optimal *ε* and *M* values for each SU node, each of which may operate in distinctive channels.

We propose a simple and pragmatic exploration approach called dynamic softmax, which is based on the traditional softmax approach. Dynamic softmax regulates the exploration temperature *M* (see (3)) of a SU based on the dynamicity and unpredictability of the network conditions. In [18], a similar approach to regulate the exploration probability of a nontraditional exploration approach is applied in wireless sensor networks.

In dynamic softmax, temperature *M* is increased and decreased by a constant factor  *f* based on the network conditions, so that more exploration is performed only when necessary (see Algorithm 1). Higher values of *f* may increase the convergence rate at the expense of higher fluctuations in SUs' network performance, whereas lower values of *f* may reduce the convergence rate; however, it achieves lower fluctuations (or higher stability) of SUs' network performance. The adjustment of temperature *M* is based on the trend of the *Q*-value of a route as follows.

- When the *Q*-value increases (i.e., higher routing cost), temperature *M* is increased in order to encourage exploration of other routes as this may indicate the emergence of PUs' activities which have degraded the SUs' network performance.
- When the *Q*-value decreases (i.e., lower routing cost), temperature *M* is left unchanged as this may indicate a forthcoming convergence to an optimal route which provides greater stability to SUs' network performance.
- When the change of *Q*-value is less than a threshold *ϑ*, the temperature *M* is decreased in order to encourage exploitation as this indicates that there has been convergence to an optimal route which provides a stable network performance.

### 5.3. Comparison of *ε*-Greedy, Softmax, Dynamic Softmax, and Exploitation-Only Approaches

This section investigates the effects of various exploration approaches applied to WCRQ-routing on network performance. We compare the network performance achieved by WCRQ-routing using two traditional exploration approaches, namely, *ε*-greedy and softmax, an exploitation-only approach and a variant of the exploration approach which we propose, namely dynamic softmax. The exploitation-only approach exploits at all times and does not explore.

While Table 3 presents the default simulation parameters and values, Table 5 presents the specific simulation parameters and values for this investigation. Generally speaking, we simplify the simulation values in order to focus on the effects of various exploration approaches on network performance in the presence of the dynamicity of the channel availability with respect to PUL, so we assume a noiseless channel with *μ*_PER_ = 0 and *σ*_PER_ = 0. The simulation results are shown for two conditions, in which the standard deviation of PUL *σ*_PUL_ ∈ {0.2, 0.8} indicates low and high levels of unpredictability of the channel availability, respectively. The exploration metrics of the traditional approaches are *ε* ∈ {0.07,0.14} and temperature *M* ∈ {0.04,0.05}, and these are chosen empirically; specifically, these are the average optimal values estimated by running extensive simulations using different values of exploration metrics under different levels of channel unpredictability *σ*_PUL_. Hence, we compare the dynamic softmax approach with the best possible network performance achieved by *ε*-greedy and softmax. For dynamic softmax (see Algorithm 1), the temperature range is [*M*_min⁡_, *M*_max⁡_] = [0.01, 0.1] because network performance degrades significantly when *M* > 0.1. The initial temperature is set to an average value of *M* = 0.05. Finally, the adjustment factor is set to *f* = 0.01 and the *Q*-value threshold is set to *ϑ* = 0.1, and these values are chosen empirically.

We present simulation results for the four performance metrics in the rest of this section.

#### 5.3.1. SUs' Interference to PUs

**Figure 11:**
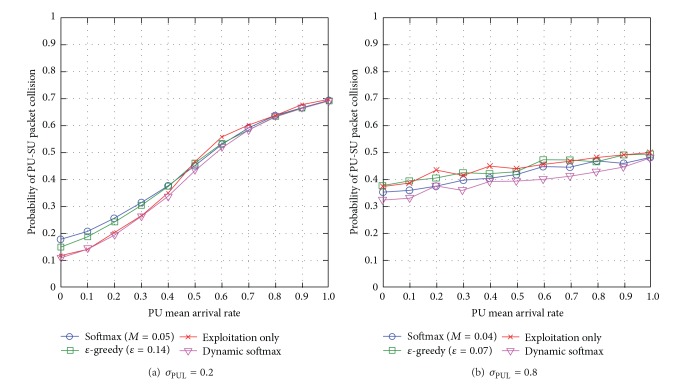
SUs' interference to PUs for varying PU mean arrival rate *μ*_PUL_ for different levels of standard deviation of PUL *σ*_PUL_.

When the standard deviation of PUL is low *σ*_PUL_ = 0.2, most next-hop node (or link) and channel pairs have very similar PU mean arrival rate *μ*_PUL_; however, dynamic softmax outperforms the traditional exploration approaches, specifically up to 39% compared to softmax and up to 22% compared to *ε*-greedy while achieving very similar network performance with the exploitation-only approach (see Figure 11(a)). When the PU mean arrival rate *μ*_PUL_ becomes higher, all channels have high levels of PUs' activities and *Q*-values among the channels do not generally vary. So, there is lack of exploration and all approaches achieve very similar network performance. When *σ*_PUL_ = 0.8, the link and channel pairs have the greatest difference in the levels of PU mean arrival rate *μ*_PUL_. Dynamic softmax outperforms the other exploration approaches, and the exploitation-only approach causes the highest SUs' interference to PUs (see Figure 11(b)). When the standard deviation of PUL *σ*_PUL_ becomes higher, all channels have different levels of PUs' activities, and *Q*-values among the channels generally vary. So, more explorations are necessary explaining why the exploitation-only approach causes the worst network performance with the highest SUs' interference to PUs. In general, softmax outperforms *ε*-greedy in most cases because softmax explores lesser below-average routes compared to *ε*-greedy.

Generally speaking, dynamic softmax achieves similar or better network performance in terms of SUs' interference to PUs compared to the traditional exploration approaches with optimal exploration metrics. This is because dynamic softmax learns the optimal exploration temperature dynamically based on the dynamicity and unpredictability levels of the network conditions. Additionally, the SUs' interference to PUs increases with the PU mean arrival rate *μ*_PUL_ and reduces with the standard deviation of PUL *σ*_PUL_.

#### 5.3.2. SU End-to-End Delay

**Figure 12:**
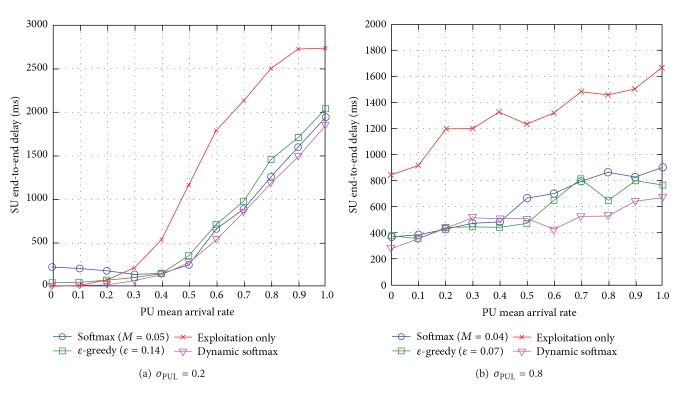
SU end-to-end delay for varying PU mean arrival rate *μ*_PUL_ for different levels of standard deviation of PUL *σ*_PUL_.

When the standard deviation of PUL is low *σ*_PUL_ = 0.2, most link and channel pairs have very similar PU mean arrival rate *μ*_PUL_. Dynamic softmax outperforms the traditional exploration approaches, and up to 52% compared to exploitation-only which incurs the highest end-to-end delay (see Figure 12(a)). The exploitation-only approach exploits the best-possible route at all times and there is lack of load balancing among routes causing network congestion and increased SU end-to-end delay. Note that when there are low levels of PU mean arrival rate *μ*_PUL_ = [0.0,0.4], softmax incurs the highest end-to-end delay because it performs the most unnecessary explorations. When *σ*_PUL_ = 0.8, the link and channel pairs have great difference in the levels of PU mean arrival rate *μ*_PUL_. Dynamic softmax outperforms the other exploration approaches (see Figure 12(b)). However, when there are low levels of PU mean arrival rate *μ*_PUL_ = [0.0,0.5], dynamic softmax incurs higher SU end-to-end delay because it performs more unnecessary explorations as a result of greater variations in *Q*-values, while *ε*-greedy explores routes randomly which increases load-balancing, and softmax chooses more above-average routes which increases network congestion.

Generally speaking, dynamic softmax reduces SU end-to-end delay compared to the traditional exploration approaches with optimal exploration metrics. Additionally, the SUs' interference to PUs increases with the PU mean arrival rate *μ*_PUL_ and reduces with the standard deviation of PUL *σ*_PUL_.

#### 5.3.3. SU Packet Loss

**Figure 13:**
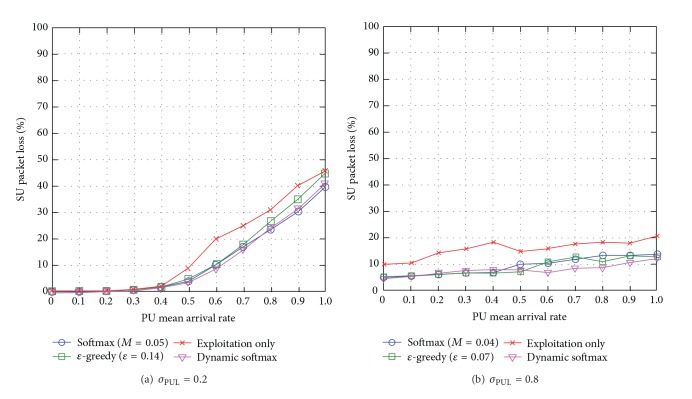
SU packet loss for varying PU mean arrival rate *μ*_PUL_ for different levels of standard deviation of PUL *σ*_PUL_.

When the standard deviation of PUL is low *σ*_PUL_ = 0.2, dynamic softmax either achieves similar network performance or outperforms the traditional exploration approaches, specifically up to 57% compared to the exploitation-only approach which incurs the highest packet loss (see Figure 13(a)). The exploitation-only approach exploits the best-possible route at all times and there is lack of load balancing among routes causing network congestion and increased SU packet loss. When *σ*_PUL_ = 0.8, dynamic softmax outperforms the other exploration approaches (see Figure 13(b)). The *ε*-greedy approach explores routes randomly which increases load-balancing, and so it outperforms softmax at times, while softmax chooses more above-average routes causing greater network congestions.

Generally speaking, dynamic softmax reduces SU packet loss compared to the traditional exploration approaches with optimal exploration metrics. Additionally, the SU packet loss increases with the PU mean arrival rate *μ*_PUL_ and reduces with the standard deviation of PUL *σ*_PUL_.

#### 5.3.4. SU Throughput

**Figure 14:**
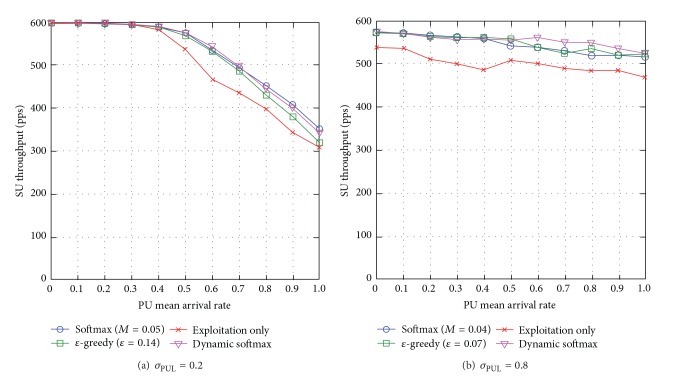
SU throughput for varying PU mean arrival rate *μ*_PUL_ for different levels of standard deviation of PUL *σ*_PUL_.

When the standard deviation of PUL is low *σ*_PUL_ = 0.2, dynamic softmax either achieves similar network performance or outperforms the traditional exploration approaches, specifically, up to 17% compared to the exploitation-only approach which incurs the highest packet loss (see Figure 14(a)). The exploitation-only approach exploits the best-possible route at all times and there is lack of load balancing among routes causing network congestion and reduced SU throughput. When *σ*_PUL_ = 0.8, similar trends are observed in network scenario of *σ*_PUL_ = 0.8 in the investigation of SU end-to-end delay (see Section 5.3.3).

Generally speaking, dynamic softmax increases SU throughput compared to the traditional exploration approaches with optimal exploration metrics. Additionally, the SU throughput reduces with the PU mean arrival rate *μ*_PUL_ and the standard deviation of PUL *σ*_PUL_.

#### 5.3.5. Section Summary

We summarize simulation outcomes for the comparison of *ε*-greedy, softmax, dynamic softmax, and exploitation-only approaches as follows.

- Network performance degrades as the dynamicity of PUs' activities *μ*_PUL_ increases.
- Exploration approaches (i.e., *ε*-greedy, softmax, and dynamic softmax) achieve load-balancing among the available routes, and hence they improve SUs' (and PUs' in some cases) network performance compared to the exploitation-only approach.
- Traditional exploration approaches, namely *ε*-greedy and softmax, outperform each other under different network conditions. For instance, when the unpredictability of the channel availability *σ*_PUL_ is low, softmax achieves better SUs' network performance than *ε*-greedy; however, when the unpredictability level of the channel availability *σ*_PUL_ becomes higher, *ε*-greedy achieves better SUs' network performance than softmax due to greater load balancing among routes, so there is lesser network congestion. Hence, neither *ε*-greedy nor softmax achieves the best-possible network performance under all network conditions.
- WCRQ-routing and CRQ-routing minimize SUs' interference to PUs with respect to PUL in the presence of dynamicity and unpredictability of the channel availability.
- Dynamic softmax achieves the best-possible PUs' and SUs' network performances in most cases compared to other approaches because it learns the best-possible exploration temperature dynamically based on the dynamicity and unpredictability levels of the channel availability.

## 6. Enhancement of Learning Rate Adjustment

The learning rate *α*, which is used to regulate the speed of convergence to the optimal or near-optimal action, is another important parameter. A suitable learning rate *α* value is essential. There have been some limited efforts to investigate the learning rate *α* in the domain of routing in wireless networks [19], and this investigation applies to routing in CRNs. We present an overview of learning rate, the traditional (called the Win-or-Learn-Fast Policy Hill Climbing approach or win-lose [6]) and our proposed (called the counterapproach) learning rate adjustment approaches to dynamically regulate the learning rate, as well as the simulation results for the four performance metrics, in the rest of this section.

### 6.1. An Overview of Learning Rate

Higher learning rate 0 ≤ *α* ≤ 1 indicates higher speed of learning and convergence rate, and it is more responsive to the dynamicity of the operating environment. Higher learning rate may cause fluctuation in *Q*-value because the *Q*-value is now more dependent on its recent estimates, which may be unstable, rather than its previous experience. On the other hand, lower learning rate may cause very low convergence rate because the *Q*-value is now more dependent on its previous experience rather than its recent estimates.

**Figure 15:**
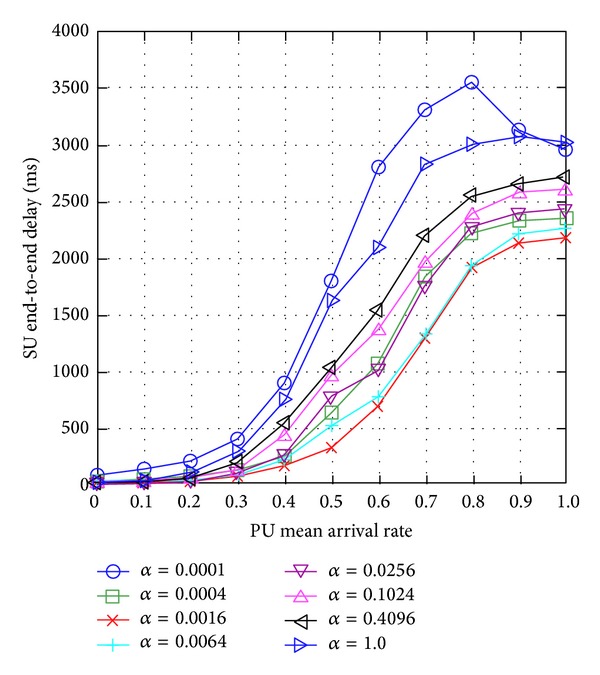
SU end-to-end delay for varying learning rate *α*.

We present simulation results of a preliminary investigation to show the effects of learning rate *α* on the SUs' network performance, particularly SU end-to-end delay. Note that more details on simulation setup and parameters are presented in later section.  Figure 15 shows that neither the lowest (i.e., *α* = 0.0001) nor the highest (i.e., *α* = 1) learning rate achieves the lowest SU end-to-end delay, and learning rate *α* = 0.0016 achieves the lowest SU end-to-end delay. Hence, too low learning rate causes a SU to learn about the available routes slowly and so it does not adapt well to the changes in the PUs' mean arrival rate, while too high a learning rate causes a SU to learn very fast and so it changes its route more frequently causing the SU end-to-end delay to increase.

Hence, a suitable learning rate *α* value is essential to provide network performance enhancement. Traditionally, the learning rate is a predefined and fixed value which is not adaptive to the dynamicity and unpredictability levels of the operating environment, and so it does not provide the best network performance in a dynamic and unpredictable operating environment.

This initial investigation motivates us to search for a suitable learning rate.

### 6.2. Learning Rate Adjustment Approaches

We present a traditional exploration approach (i.e., the win-lose approach) and a variant of the exploration approach (i.e., the counterapproach), as well as a baseline approach (i.e., the random approach), in this section.

#### 6.2.1. The Win-Lose Approach

In [20], a stochastic learning algorithm, namely, win-or-learn-fast policy hill climbing (or win-lose for simplicity) [6] is applied to regulate the learning rate dynamically based on the dynamicity of the operating environment in a wireless network. Given that higher *Q*-values provide network performance enhancement, the algorithm defines* winning* and* losing* as receiving higher and lower *Q*-values than its current *Q*-value, which is its expectation, respectively. When the algorithm is winning, the learning rate is set to a lower value, and vice-versa. The reason is that when a SU wins, it must be cautious in changing its routing policy and more time must be given to other SUs to adjust their own policies in favor of this winning. On the other hand, when a SU node loses, it must adapt faster to changes in the operating environment because its network performance (or rewards) is lower than its expectation.

#### 6.2.2. The Counterapproach

The traditional win-lose approach (see Section 6.2.1) regulates the learning rate for each update of *Q*-value. Nevertheless, in a highly dynamic and unpredictable operating environment, *Q*-values may vary greatly and this causes frequent changes to learning rate. As a consequence, there are frequent changes to routing decision causing higher fluctuation in SUs' network performance.

The proposed counterapproach addresses the aforementioned issue by regulating the learning rate based on the historical *Q*-values. Specifically, for each *Q*-value (or state-action pair), it keeps track of a counter for winning *c*_*t*_^*p*^ and another counter for losing *c*_*t*_^*n*^ (see Algorithm 2). Subsequently, it calculates a ratio *o*_*t*_^*n*^ = *c*_*t*_^*p*^/*c*_*t*_^*n*^, which represents the number of winning to the number of losing for a *Q*-value. When the ratio is *o*_*t*_^*n*^ > 1, the learning rate is set to a lower value because this indicates a winning event, while the learning rate is set to a higher value when the ratio is *o*_*t*_^*n*^ < 1 because this indicates a losing event, and when the ratio is *o*_*t*_^*n*^ = 1, the learning rate is unchanged as it indicates a stable network performance. Similarly, when the newly received *Q*-value is similar to the previous value, the counter is not updated. Note that we assume the learning rate *α* is increased and decreased by a small constant factor  *f* in order to avoid fluctuations in SUs' network performance.

#### 6.2.3. The Random Approach

The random approach, which does not apply any learning mechanism, regulates the learning rate in a round robin fashion without considering the dynamicity and unpredictability of the operating environment. This scheme serves as a baseline for comparison with win-lose and the counterapproach, and more importantly, it is used to show the effects of nonoptimal learning rates on network performance. This approach regulates the learning rate whenever it loses. Specifically, an agent decreases its learning rate *α* until the minimum learning rate limit *α*_min⁡_ is reached, and then it increases the learning rate *α* again until the maximum learning rate limit *α*_max⁡_  is reached, and this continues to loop in between the minimum *α*_min⁡_ and the maximum *α*_max⁡_ limits.

### 6.3. Comparison of Win-Lose and Counter and Random Approaches

This section investigates the effects of various learning rate adjustment approaches applied to WCRQ-routing on network performance. We compare the network performance achieved by WCRQ-routing using a traditional learning rate adjustment approach (i.e., win-lose), a variant of the win-lose (i.e., the counterapproach), a baseline (i.e., the random approach), and another baseline that provides the best empirical learning approach (which we may conveniently call* best*). The best empirical approach provides the best possible network performance, and the learning rate *α* is obtained by running extensive simulations under different levels of channel unpredictability.

While Table 3 presents the default simulation parameters and values, Table 6 presents the specific simulation parameters and values for this investigation. Generally speaking, we simplify the simulation values in order to focus on the effects of various learning rate adjustment approaches on network performance in the presence of the dynamicity of the channel availability with respect to PUL, so we assume a noiseless channel with *μ*_PER_ = 0 and *σ*_PER_ = 0. The simulation results are shown for two conditions, in which the standard deviation of PUL *σ*_PUL_ ∈ {0.2, 0.8} indicates low and high levels of the unpredictability of channel availability, respectively. The best empirical learning rate is set to *α* ∈ {0.0064, 0.004}, which is estimated by running extensive simulations. The range of the learning rate is [*α*_min⁡_, *α*_max⁡_] = [0.001, 0.1] because network performance degrades significantly when *α* > 0.1. Finally, the learning rate adjustment factor is *f* = 0.001, which is estimated by running extensive simulations with the objective of enhancing SUs' network performance.

We present simulation results for the four performance metrics in the rest of this section.

#### 6.3.1. SUs' Interference to PUs

**Figure 16:**
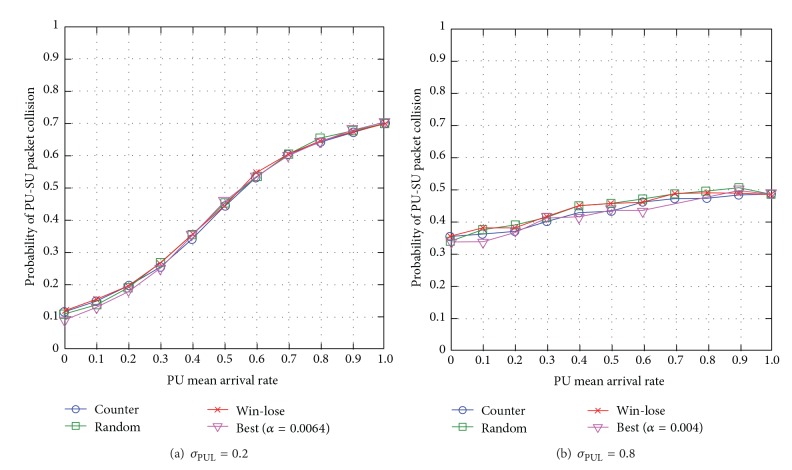
SUs' interference to PUs for varying PU mean arrival rate *μ*_PUL_ for different levels of standard deviation of PUL *σ*_PUL_.

When the standard deviation of PUL is low *σ*_PUL_ = 0.2, most next-hop node (or link) and channel pairs have very similar PU mean arrival rate *μ*_PUL_. The effect of learning rate on network performance is minimal and so all the learning rate adjustment approaches achieve almost similar network performance (see Figure 16(a)). When *σ*_PUL_ = 0.8, the link and channel pairs have great difference in the levels of PU mean arrival rate *μ*_PUL_, and the* Q*-values among the channels generally vary greatly. The counterapproach achieves almost similar SUs' interference to PUs compared to the best empirical approach and the lowest interference level compared to the win-lose and random approaches (see Figure 16(b)). This is because when *σ*_PUL_ is high, the counterapproach chooses a suitable learning rate that reduces fluctuations in* Q*-values while making routing decisions, while the win-lose and random approaches adjust the SU learning rate very fast resulting in more frequent changes to SU route decision, and hence higher SUs' interference to PUs.

Generally speaking, the counterapproach reduces SUs' interference to PUs. Additionally, the SUs' interference to PUs increases with the PU mean arrival rate *μ*_PUL_ and reduces with the standard deviation of PUL *σ*_PUL_.

#### 6.3.2. SU End-to-End Delay

**Figure 17:**
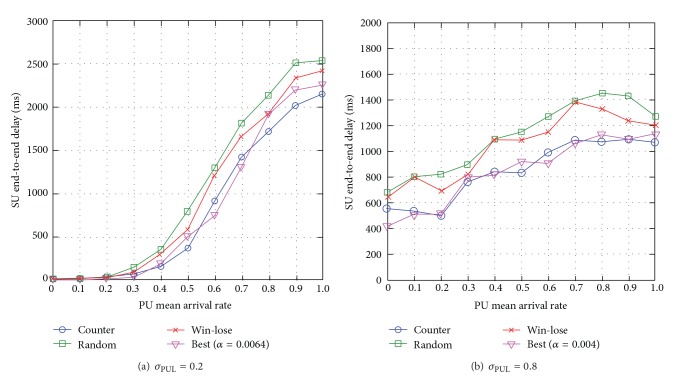
SU end-to-end delay for varying PU mean arrival rate *μ*_PUL_ for different levels of standard deviation of PUL *σ*_PUL_.

When the standard deviation of PUL is low *σ*_PUL_ = 0.2, most link and channel pairs have very similar PU mean arrival rate *μ*_PUL_. The counterapproach achieves almost similar SU end-to-end delay compared to the best empirical approach and the lowest SU end-to-end delay compared to the win-lose and random approaches, specifically up to 11% compared to win-lose which incurs the highest packet loss (see Figure 17(a)). The random approach has the highest SU end-to-end delay, which is up to 12% higher compared to the best empirical learning rate approach, and this shows the effects of non-optimal learning rate on SU end-to-end delay. When *σ*_PUL_ = 0.8, the link and channel pairs have great difference in the levels of PU mean arrival rate *μ*_PUL_, and the* Q*-values among the channels generally vary greatly. So, the learning rate has greater effects on SU end-to-end delay as a SU needs a suitable learning rate to learn about the available routes while minimizing fluctuations in *Q*-values. Similarly, the counterapproach achieves almost similar SU end-to-end delay to PUs compared to the best empirical approach, and the lowest SU end-to-end delay compared to the win-lose and random approaches, specifically up to 21% lower compared to win-lose (see Figure 17(b)). This is because when *σ*_PUL_ is high, the counterapproach chooses a suitable learning rate that reduces fluctuations in *Q*-values while making routing decisions, while the win-lose and random approaches adjust the SU learning rate very fast resulting in more frequent changes to SU route decision, and hence higher SU end-to-end delay. When PU mean arrival rate is *μ*_PUL_ = 1, the win-lose and random approaches reduce SU end-to-end delay since faster adjustment of learning helps to achieve load balancing among the available routes.

Generally speaking, the counterapproach reduces SU end-to-end delay. Additionally, the SU end-to-end delay increases with the PU mean arrival rate *μ*_PUL_ and reduces with the standard deviation of PUL *σ*_PUL_.

#### 6.3.3. SU Packet Loss

**Figure 18:**
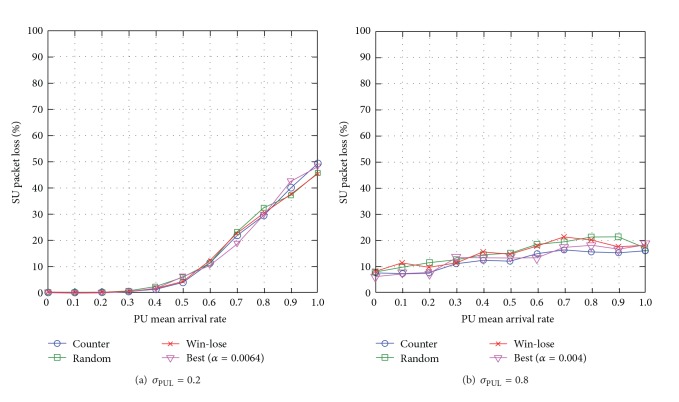
SU packet loss for varying PU mean arrival rate *μ*_PUL_ for different levels of standard deviation of PUL *σ*_PUL_.

When the standard deviation of PUL is low *σ*_PUL_ = 0.2, the effect of learning rate is minimal and so all the learning rate adjustment approaches achieve almost similar network performance. When the PU mean arrival rate is *μ*_PUL_ = [0.8, 1.0], the win-lose and random approaches achieve lower packet loss compared to other approaches, specifically up to 8% lower compared to the counterapproach (see Figure 18(a)). This is because the win-lose and random approaches adjust the SU learning rate faster than the counterapproach leading to more frequent changes to routes, and as the routes become more congested, this helps to achieve load balancing among the available routes and reduces SU packet loss. When *σ*_PUL_ = 0.8, the learning rate has slightly greater effects on SU packet loss as a SU needs a suitable learning rate to learn about the available routes while minimizing fluctuations in *Q*-values. Similarly, the counterapproach achieves almost similar SU packet loss compared to the best empirical approach, and the lowest SU packet loss compared to the win-lose and random approaches, specifically up to 23% lower compared to win-lose (see Figure 18(b)). This is because when *σ*_PUL_ is high, the counterapproach chooses a suitable learning rate that reduces fluctuations in* Q*-values while making routing decisions.

Generally speaking, the counterapproach reduces SU packet loss. Additionally, the SU packet loss increases with the PU mean arrival rate *μ*_PUL_ and reduces with the standard deviation of PUL *σ*_PUL_.

#### 6.3.4. SU Throughput

**Figure 19:**
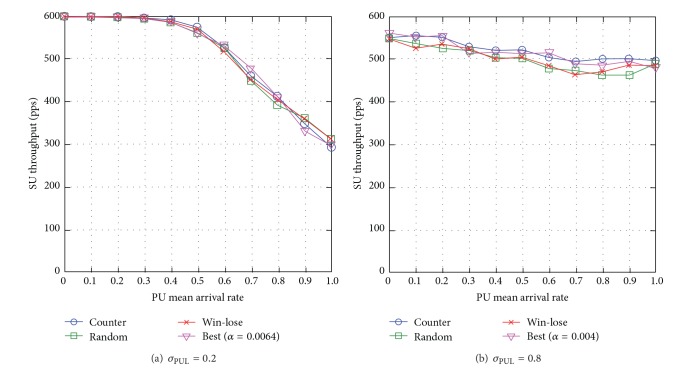
SU throughput for varying PU mean arrival rate *μ*_PUL_ for different levels of standard deviation of PUL *σ*_PUL_.

When the standard deviation of PUL is low *σ*_PUL_ = 0.2, the effect of learning rate is minimal and so all the learning rate adjustment approaches achieve almost similar network performance (see Figure 19(a)), and the reasons are similar to those observed in the investigation of SU packet loss (see Section 6.3.3). When *σ*_PUL_ = 0.8, the counterapproach achieves almost similar SU throughput compared to the best empirical approach, and the highest SU throughput compared to the win-lose and random approaches, specifically up to 7% higher compared to win-lose (see Figure 19(b)), and the reasons are similar to those observed in the investigation of SU packet loss (see Section 6.3.3).

Generally speaking, the SU throughput decreases with the PU mean arrival rate *μ*_PUL_ and reduces with the standard deviation of PUL *σ*_PUL_.

#### 6.3.5. Section Summary

We summarize simulation outcomes for the comparison of win-lose, counter and random approaches as follows.

- Network performance degrades as the dynamicity of PUs' activities *μ*_PUL_ increases.
- The effects of learning rate on SUs' network performance increase with the unpredictability of the PUs' activities *σ*_PUL_.
- When the PU mean arrival rate is high (i.e., *μ*_PUL_ = [0.8, 1.0]), where route congestion is higher, the win-lose and random approaches improve SUs' network performance. This is because these approaches regulate the learning rate at higher speed, and this helps to achieve load balancing among the available routes, and subsequently enhances SUs' network performance.
- The counterapproach achieves almost similar SU network performance to the best empirical learning rate approach and outperforms the win-lose and random approaches in most cases, particularly when the unpredictability of the PUs' activities *σ*_PUL_ becomes higher. This is because the counterapproach chooses a suitable learning rate to reduce fluctuations in* Q*-values which start to vary at higher *σ*_PUL_ values.

## 7. Conclusions

Through simulation, this paper investigates the effects of reinforcement learning (RL) parameters on network performance for routing scheme in the presence of the dynamicity and unpredictability of the channel availability in cognitive radio ad hoc networks. We present WCRQ-routing that incorporates a weight factor *ω* in the reward representation to adjust the tradeoff between PUs' and SUs' network performances, as well as to further improve the overall network performance of SUs. Higher weight factor *ω* increases PUs' network performance, while lower *ω* increases SUs' network performance, and so a balanced value of *ω* helps to achieve the best SUs' network performance, particularly lower packet loss and higher throughput. The SUs' network performance can be further enhanced by regulating exploration probability and learning rate. We present a simple and pragmatic exploration approach called* dynamic softmax* to regulate the exploration temperature. Dynamic softmax learns a near-optimal exploration temperature dynamically according to the dynamicity and unpredictability of the channel availability, and it achieves better network performance in most cases compared to the traditional exploration approaches, namely *ε*-greedy and softmax. We present a simple and pragmatic learning rate adjustment approach called the* control* approach to regulate the learning rate dynamically based on the historical *Q*-values. The counterapproach achieves better SUs' network performance compared to the traditional win-lose approach, and it achieves almost similar SUs' network performance to the best empirical learning rate approach by learning a near-optimal learning rate dynamically based on the dynamicity and unpredictability of the operating environment.

## Figures and Tables

**Algorithm 1:**
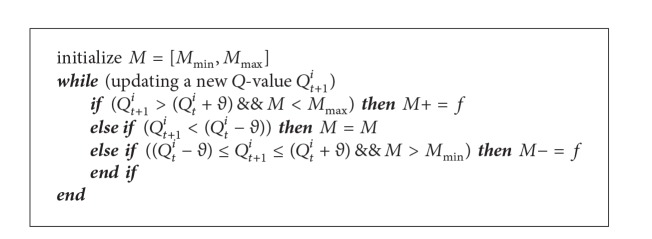
Dynamic softmax algorithm at SU node *i*.

**Algorithm 2:**
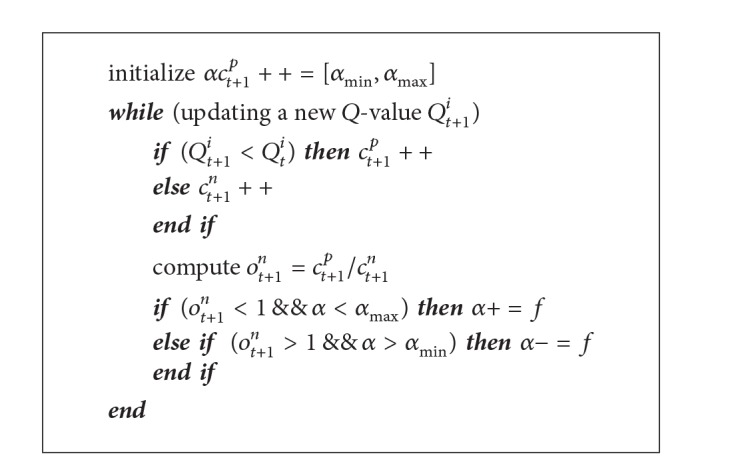
Counterapproach algorithm at SU node *i*.

**Table 1:** CRQ-routing model embedded at SU node *i*.

State	*s*_*t*_^*i*^ ∈ *S* = {1,2,…, *N* − 1}, each state *s*_*t*_^*i*^ representing a SU destination node *n*. *N* represents the number of SUs in the entire network.

Action	*a*_*t*_^*i*^ ∈ *A* ^*i*^ = {1,2,…, *J*}, each action *a*_*t*_^*i*^ representing the selection of a SU next-hop node *j* along with its operating channel. *J* represents the number of SU *i*'s neighboring SU nodes.

Cost	*r*_*t*_^*i*^(*a*_*t*_^*i*^) represents the link-layer delay incurred to successfully deliver a packet from SU node *i* to SU neighbor node *a*_*t*_^*i*^ = *j*, including retransmission delays as a result of PU-SU packet collision and packet loss.

**Table 2:** Reward representation for WCRQ-routing model embedded at SU node *i*.

Cost	*r*_*t*_^*i*^(*a*_*t*_^*i*^) = *ωr*_*t*_^*i*,*j*^ + (1 − *ω*)*q*_*t*_^*j*^ where *r*_*t*_^*i*,*j*^ represents the number of retransmissions for a packet sent from SU node *i* to SU neighbor node *j* at time *t*, while *q*_*t*_^*j*^ represents the number of packets in the queue of SU neighbor node *j*. Weight factor *ω* = [0,1] is used to adjust the tradeoff between PUs' and SUs' network performance.

**Table 3:** Simulation parameters and values.

Category	Parameter	Value
SU	SU's transmission delay, *d*_SU_^tr⁡^	1.0 ms
	Processing delay, *d*_SU_^pr^	1.0 ms
	Mean arrival rate, *λ*_SU_	0.6
	Learning rate, *α*	0.5
	WCRQ-routing weight factor, *ω*	0.5

PU	PU's transmission delay, *d*_PU_^tr⁡^	1.2 ms
	Mean arrival rate (or PUL), *μ*_PUL_	[0.0, 1.0]
	Standard deviation, *σ*_PUL_	{0.0, 0.4, 0.8}

Channel	Mean PER, *μ*_PER_	0.05
	Standard deviation of PER, *σ*_PER_	0.025

**Table 4:** Simulation parameters and values for investigating the effects of weight factor in reward representation.

Category	Parameter	Value
SU	Mean arrival rate, *λ*_SU_	0.8

PU	Mean arrival rate (PUL), *μ*_PUL_	{0.2, 0.4}
	Standard deviation, *σ*_PUL_	0.5

Channel	Mean PER, *μ*_PER_	{0.2, 0.4}
	Standard deviation of PER, *σ*_PER_	0.2

**Table 5:** Simulation parameters and values for investigating the exploration approaches.

Category	Parameter	Value
SU	Traditional *ε*-greedy exploration probability, *ε*	{0.07, 0.14}
	Traditional softmax exploration temperature, *M*	{0.04, 0.05}
	Initial dynamic softmax temperature, *M*	0.05
	Dynamic softmax adjustment factor, *f*	0.01
	Dynamic softmax temperature range, [*M*_min⁡_, *M*_max⁡_]	[0.01, 0.1]
	Dynamic softmax *Q*-value threshold, *ϑ*	0.1

PU	Standard deviation of PUL, *σ*_PUL_	{0.2, 0.8}

Channel	Mean PER, *μ*_PER_	0
	Standard deviation of PER, *σ*_PER_	0

**Table 6:** Simulation parameters and values for investigating the learning rate adjustment approaches.

Category	Parameter	Value
SU	Best empirical learning rate, *α*	{0.0064, 0.004}
	Learning rate adjustment factor, *f*	0.001
	Learning rate range, [*α*_min⁡_, *α*_max⁡_]	[0.001, 0.1]

PU	Standard deviation of PUL, *σ*_PUL_	{0.2, 0.8}

Channel	Mean PER, *μ*_PER_	0
	Standard deviation of PER, *σ*_PER_	0
